# Assessing failure of transfer of passive immunity by gamma-glutamyl-transferase activity and serum refractometry in holstein-friesian calves affected by neonatal diarrhea

**DOI:** 10.1007/s11259-023-10149-3

**Published:** 2023-06-14

**Authors:** Giulia Sala, Valerio Bronzo, Antonio Boccardo, Alessia Libera Gazzonis, Pierangelo Moretti, Vincenzo Ferrulli, Angelo Giovanni Belloli, Laura Filippone Pavesi, Gaia Pesenti Rossi, Davide Pravettoni

**Affiliations:** Department of Veterinary Medicine and Animal Science (DIVAS), University of Milan, Via dell’Università 6, Lodi, 26900 Italy

**Keywords:** Neonatal calf diarrhea, Passive immunity, Serum total protein, Gamma-glutamyl-transferase activity

## Abstract

Hardly published data are available to diagnose the failure of transfer of passive immunity (FTPI) in calves affected by neonatal calf diarrhea (NCD). This study evaluated the diagnostic performance and differences among optical serum total protein (STP) concentration and gamma-glutamyl-transferase (GGT) activity for assessing FTPI in diarrheic Holstein Friesian calves. 72 diarrheic and 19 healthy Holstein Friesian calves aged 1 to 10 days were enrolled. Each calf underwent a complete clinical examination and dehydration assessment. The effect of dehydration status and age on the correlation between the two methods under study (STP and GGT) and the gold standard (Immunoglobulin G [IgG] measured with RID) was investigated with Spearman’s correlation index R for ranks. Serum total protein concentration and GGT activity were analyzed by receiver operating characteristic (ROC) curve analysis to identify the optimal cut-off point to distinguish between diarrheic calves with or without FTPI, also considering the effects of dehydration and age. The results show that GGT activity was affected by the age of calves, while STP was influenced by dehydration. The cut-offs to distinguish calves with IgG < 10 g/L were < 52 g/L of STP in normohydrated calves, < 58 g/L of STP in dehydrated calves, and < 124 IU/L of GGT in calves with aged between 3 and 10 days. In nondehydrated diarrheic calves, the STP refractometer showed better diagnostic accuracy.

In dehydrated calves aged between 3 and 10 days, however, its accuracy drops, and it is advisable to use GGT activity.

**Supplementary Information:**

The online version contains supplementary material available at 10.1007/s11259-023-10149-3.

## Introduction

The incidence of neonatal calf diarrhea (NCD) is often directly correlated with failure of transfer of passive immunity (FTPI) (Lora et al. 2018) and is characterized by fluid losses, increased inflammatory proteins, or hypoproteinemia, which complicate the indirect assessment of the immune status of affected calves (Tyler et al. 1999; Buczinski et al. 2018; Athanasiou et al. 2019). A cost-effective indirect method to evaluate FTPI in diarrheic calves may be attractive in optimizing treatment protocol or evaluating colostrum management practices during an NCD outbreak. Serum gamma-glutamyl-transferase (GGT) activity is a good indicator of colostrum ingestion and is minimally affected by hydration status (Braun et al. 1982; Mugnier et al. 2020). Although serum refractometry is a more accessible technique with fewer limitations and higher accuracy than GGT in healthy calves (Zakian et al. 2018; Cuttance et al. 2019; Hue et al. 2021), in sick calves, GGT activity showed a good correlation with serum Immunoglobulin G (IgG) concentration. Tyler et al. (1999) found that GGT activity was strongly correlated with total serum protein (STP) concentration for indirectly assessing serum IgG concentration in critically ill calves. Similarly, Fecteau and colleagues (2013) confirmed the high positive and negative predictive value of GGT activity compared to the reference radial immunodiffusion (RID) test with a higher correlation coefficient than the STP in sick Holstein calves.

Currently, however, there are no data regarding the diagnostic performance of serum refractometry and GGT activity in diarrheic calves without additional comorbidities and considering the severity of the clinical presentation. Thus, this study aimed to evaluate the diagnostic performance of optical STP refractometer and serum GGT activity in diarrheic Holstein calves for serum IgG estimation measured with the reference RID test considering the influence of dehydration status and age.

## Materials and methods

A diagnostic accuracy study was performed according to guidelines for reporting diagnostic accuracy (Bossuyt et al. 2015), using clinical records of diarrheic client-owned Holstein Friesian calves admitted to the Veterinary Teaching Hospital of the University of Milan and a convenient sample of healthy Holstein Friesian calves from the same farms that referred NCD calves on our clinic during an outbreak of disease and checked by our ambulatory service between May 2018 and May 2020.

Hospitalized calves were considered affected by NCD when presented with loose or watery feces, corresponding to a fecal score of 2 or 3 on a 0 to 3 scale, as reported by McGuirk (2008). Within this sample, we considered diarrheic Holstein Friesian females and males that had undergone a blood gas analysis, an evaluation of STP by hand refractometer, and for which a residual serum sample was stored at -20 °C on the day of hospitalization. Other concurrent diseases at the time of hospitalization represented an exclusion criterion. Only calves, aged between 1 and 10 days were enrolled to avoid distorting the correlation between serum refractometry and colostral IgG using older calves (Wilm et al. 2018). Based on previous investigations, clinical scores were used to analyze dehydration status (Boccardo et al. 2017, 2019). The dehydration score was estimated according to the following scoring system: score (1) normal hydration, upper eyelid skin tent < 2 s; score (2) moderate dehydration, eyeball slightly sunken (1–2 mm), and upper eyelid skin tent > 2 s but < 4 s (estimated loss of body mass 3–5%); score (3) noticeable dehydration, sunken eyes (3–4 mm), dry nose, upper eyelid skin tent > 5 s (estimated loss of body mass 6–8%); score (4) severe sunken eyes with an easily perceptible distance between the eyeball and the eyelid (≥ 5 mm), cold ears, legs, and oral cavity, dry mouth, and nose, upper eyelid skin tent persist (estimated loss of body mass ≥ 9%). For each diarrheic calf, the blood sample was obtained at admission and before any treatment was administered in the clinic, so the treatment could not affect the laboratory results. In healthy animals, blood sampling was performed during the farm visit to determine the transfer of passive immunity, as requested by the owner. The data obtained were used for this study. For the control group, calves were considered healthy based on history and clinical examination. Serum IgG was determined by RID using a bovine IgG commercial kit (Bovine IgG Test Kit Radial Immunodiffusion Test Kit, Triple J Farms, Washington, USA) according to the manufacturer’s instructions and analyzed within one month from sampling for all serum samples. All the sera samples were analyzed in a single batch, as indicated by the manufacturer. Test results were compared with a standard curve prepared using bovine immunoglobulin standards supplied with the Kit. The STP concentration was measured by a handheld refractometer (mod. MR514ATC, Milwaukee Srl, Gallarate, Italy), calibrated before measurement, and made by the same operator (first author, GS) to prevent operator-dependent bias. Gamma-glutamyl transferase (GGT) activity was determined using an automated spectrophotometer (BT 3500, Biotecnica instruments, Roma, Italy), and reagents were provided by Futurlab Srl (Limena, Padova). Quality controls were performed daily with two levels of human-based sera provided by the manufacturer of the instrument (“Control N” and “Control P”; Biotecnica Instruments, Rome, Italy).

Data storage, descriptive statistics and non-parametric analysis were performed with IBM SPSS Statistics v. 27.0 (IBM Corp., Armonk, NY). The quantitative variables collected were age, IgG, STP, and GGT concentration, while the qualitative variables were sex, presence/absence of NCD and degree of dehydration. In addition, three qualitative variables were calculated:

1. Four classes for IgG level, according to the classification of Lombard et al. (2020): excellent (> 25 g/L), good (18-24.9 g/L), fair (10-17.9 g/L), and poor (< 10 g/L);
2. Two age classes: ≤ 3 days and > 3 days, for the influence of age on GGT activity, according to Braun et al. (1982), to distinguish between the peak phase, which is characterized by an inter-individual substantial variability that usually occurs during the first 48 days of life and the slight decrease that occurs after the peak within the first 20 days of life;
3. Presence and absence of dehydration.

Data were not normally distributed, assessed by the Shapiro-Wilk test. Then, the quantitative variables were reported with the median, 1st, and 3rd quartile (Q1 and Q3), and qualitative variables were reported with frequency and percentage.

Interactions between the variables under investigation were analyzed with non-parametric tests. The median of IgG, STP, and GGT activity in calves affected or unaffected by NCD was compared with the U Mann – Whitney test. The effect of dehydration and categorized IgG on STP and GGT concentration were investigated with the Kruskal-Wallis test. The correlation between IgG and STP concentration and IgG and GGT activity was analyzed with Spearman’s correlation index R for ranks. These correlations were investigated in the whole sample and only in animals with NCD. In addition, based on the results of non-parametric tests, the correlation between IgG and STP was investigated separately in nondehydrated and dehydrated animals, and the correlation between IgG and GGT was investigated in the different age groups. Bonferroni’s correction was used to account for multiple comparisons.

The accuracy analysis of diagnostic tests was performed with MedCalc® Statistical Software version 22.003 (MedCalc Software Ltd, Ostend, Belgium; https://www.medcalc.org; 2023). Cut-offs of STP and GGT in NCD animals were determined with the Receiver Operating Characteristic (ROC) curve for different classes of IgG (< 10 g/L; 18 g/L, and < 25 g/L), considering factors affecting the correlation between IgG, STP, and GGT. Cut-offs were chosen using the Youden index (J) (Youden, 1950), where sensitivity and specificity are maximized, and equal weight is given to false-positive and false-negative results (J = Sensitivity + Specificity – 1). In addition, the areas under the curve (AUC) and their 95% confidence intervals (CI) were calculated and used as indicators of test accuracy to discriminate the IgG level of calves. The interpretation of AUC is based on a 1.00 perfect test, 0.99 − 0.9 excellent test, 0.89 − 0.80 good test, 0.79 − 0.70 fair test, 0.69 − 0.51 poor test, 0.50 failed test (Hanley and McNeil 1982). The cut-off values selected were used to estimate sensitivity (Se), specificity (Sp), positive predictive value (PPV), negative predictive value (NPV), and accuracy for both STP and GGT.

A posthoc power analysis based on the Wilcoxon-Mann-Whitney test was performed to estimate the minimal differences between groups (NCD vs. healthy; normohydrated vs. dehydrated; FTPI vs. not FTPI); The power in each sample categorization was > 80%. The power analysis was performed using G*Power (Ver. 3.1, Heinrich-Heine-Universität, Düsseldorf, Germany).

## Results

Ninety-one calves were included in the study. Of these, 72 (79.1%) calves were affected by NCD, while 19 (28.9%) were healthy animals. In the sample, 12 (13.2%) were males (10 with NCD and 2 healthy), and 79 (86.2%) were females (62 with NCD and 17 healthy). Calves with NCD and concomitant dehydration were 54 (75%), while 18 (25%) had no dehydration despite the disease. Among the dehydrated animals, 20 (37%) animals presented 3–5% fluid loss, 27 (50%) calves about 6–8%, and 7 (13%) animals ≥ 9%. No healthy calves had dehydration. The percentages of calves in the IgG concentration classes (Lombard et al. 2020) were shown in supplementary Table 3.

The difference in IgG concentration (healthy calves: median 25.4, Q1 18.9 and Q3 28.6 g/L; NCD calves: median 14.2, Q1 9.4 and Q3 21.9 g/L; *P* 0.009), STP concentration (healthy calves: median 54, Q1 52 and Q3 58 g/L; NCD calves: median 60, Q1 52 and Q3 68 g/L; *P* 0.040), and age (healthy calves: median 7, Q1 4 and Q3 8 days; NCD calves: median 8, Q1 5 and Q3 10 days; *P* 0.042) in healthy and NCD calves were statistically significant, while the difference in GGT activity was not statistically significant (healthy calves: median 234, Q1 106 and Q3 525 IU/L; NCD calves: median 158, Q1 84.5 and Q3 343 IU/L; *P* 0.081) (Supplementary files: supplementary Tables 1, supplementary Tables 2 and Fig. 1).

Age classes only significantly affected gamma-glutamyl-transferase activity (calves ≤ 3 days: 470 IU/L, calves > 3 days: 156 IU/L P 0.003). While STP concentration in the whole sample was only significantly affected by dehydration (normohydrated calves: 55 g/L, dehydrated calves: 60 g/L; *P* 0.013). In animals with NCD, multiple comparisons showed that only the STP concentration of 6–8% dehydrated calves differed from normohydrated ones (normohydrated calves: 58 g/L, 6–8% dehydrated calves: 66 g/L; *P* 0.004). According to the classification of IgG by Lombard et al. (2020), the concentration of STP and GGT are statically different (*P* < 0.001 and 0.025, respectively). The multiple comparisons showed that the difference in STP and GGT was statistically significant between the IgG poor category and others (Supplementary file: supplementary Table 4).

The correlation between IgG and STP in the whole sample had a Spearman’s coefficient of 0.53 (*P* < 0.001), while the correlation between IgG and GGT had a Spearman’s coefficient of 0.63 (*P* < 0.001). The influence of NCD, dehydration, and age on these correlations is shown in Table 1. Table 2 shows the calculated AUC and cut-offs from the ROC curves analysis (Supplementary files: Figures 2 and 3). As the table highlights, the best test for assessing the IgG level is STP, but only in normohydrated calves. GGT activity is an excellent test for discriminating calves with IgG < 10 g/L and age > 3 days, while in dehydrated calves, STP is a good test to differentiate calves with IgG < 10 g/L. The calculated diagnostic accuracy indices for the calculated cut-offs are shown in Table 3.

**Table 1:** Spearman’s coefficient for correlations between immunoglobulin G (IgG), serum total protein (STP), and gamma-glutamyl transferase (GGT), divided for the variables neonatal calf diarrhea (NCD), presence/absence of dehydration and age in Holstein Friesian calves

Correlation	Classification variable	Spearman’s coefficient
IgG-STP	NCD (n = 91)	Healthy calves: 0.73 (*P* 0.004)
		Calves with NCD: 0.65 (*P* < 0.001)
IgG-GGT	NCD (n = 91)	Healthy calves: 0.59 (*P* 0.007)
		Calves with NCD: 0.63 (*P* < 0.001)
IgG-STP	Dehydration (only calves with NCD) (n = 72)	Normohydrated calves: 0.90 (*P* < 0.001)
		Dehydrated calves: 0.63 (*P* < 0.001)
IgG - GGT	Age (only calves with NCD) (n = 72)	Age ≤ 3 days: 0.48 (*P* 0.065)
		Age > 3 days: 0.71 (*P* < 0.001)
IgG-GGT	Dehydration (only calves with NCD) (n = 72)	Normohydrated calves: 0.68 (*P* < 0.001)
		Dehydrated calves: 0.68 (*P* < 0.001)

**Table 2:** Optimal criteria (cut point) in 72 calves with neonatal calf diarrhea (NCD) determined by receiver operating characteristic (ROC) curves for assessing the failure of transfer of passive immunity (FTPI) for serum total protein concentration (STP) in both normohydrated and dehydrated patients and gamma-glutamyl-transferase activity (GGT) in calves with age > 3 days, using three different cut-off points (serum immunoglobulin G concentration < 10 g/L, 18 g/L, and 25 g/L)

Cut-off value for diagnosing FTPI	Classification variable	N	J	Cut point	AUC	CI 95%
IgG: 10 g/L	STP in normohydrated calves	18	0.75	56 g/L	0.92	0.70-1.00
	STP in dehydrated calves	54	0.59	56 g/L	0.82	0.70–0.92
	GGT in calves with age > 3 days	61	0.78	124 IU/L	0.94	0.84–0.98
	GGT in normohydrated calves > 3 days	15	0.90	147 IU/L	0.92	0.66-1.00
	GGT in dehydrated calves > 3 days	46	0.88	100 IU/L	0.95	0.84–0.95
IgG: 18 g/L	STP in normohydrated calves	18	0.75	56 g/L	0.92	0.69–0.99
	STP in dehydrated calves	54	0.37	65 g/L	0.73	0.59–0.84
	GGT in calves with age > 3 days	61	0.58	147 IU/L	0.80	0.68–0.89
	GGT in normohydrated calves > 3 days	15	0.67	147 IU/L	0.85	0.58–0.98
	GGT in dehydrated calves > 3 days	46	0.55	142 IU/L	0.80	0.65–0.90
IgG: 25 g/L	STP in normohydrated calves	18	1.00	60 g/L	1.00	0.82-1.00
	STP in dehydrated calves	54	0.38	75 g/L	0.74	0.60–0.85
	GGT in calves with age > 3 days	61	0.51	147 IU/L	0.77	0.65–0.87
	GGT in normohydrated calves > 3 days	15	0.62	185 IU/L	0.73	0.44–0.92
	GGT in dehydrated calves > 3 days	46	0.54	142 IU/L	0.79	0.64–0.91

AUC, area under the curve; CI 95%, 95% confidence interval; J, Youden index (J = Sensitivity + Specificity – 1)

**Table 3:** Diagnostic test characteristics for serum total protein concentration (STP) in both normohydrated and dehydrated patients and gamma-glutamyl-transferase activity (GGT) in calves with age > 3 days for assessing the failure of transfer of passive immunity in 72 diarrheic Holstein Friesian calves using three different cut-off points (serum immunoglobulin G concentration 10 g/L, 18 g/L, and 25 g/L)

Cut-off value for diagnosing FTPI	Classification variable	N	Se (%) (CI 95%)	Sp (%) (CI 95%)	PPV (%) (CI 95%)	NPV (%) (CI 95%)	Acc (%) (CI 95%)
IgG 10 g/L	STP in normohydrated calves	18	100.0 (54.1–100.0)	75.0 (42.8–94.5)	66.7 (42.9–84.2)	100.0	83.3 (58.6–96.4)
	STP in dehydrated calves	54	75.0 (47.6–92.7)	84.2 (78.7–94.0)	66.7 (47.7–81.5)	88.9 (77.2–95.0)	81.5 (68.6–90.8)
	GGT in calves with age > 3 gg	61	94.7 (74.0 -99.9)	83.3 (68.6–93.0)	72.0 (56.5–83.6)	97.2 (83.8–99.6)	85.3 (73.8–93.0)
	GGT in normohydrated calves > 3 days	15	100.0 (47.8–100.0)	90.0 (55.5–99.7)	83.3 (43.8–97.0)	100.0	93.3 (68.1–99.8)
	GGT in dehydrated calves > 3 days	46	100.0 (76.8–100.0)	87.5 (71.0 -96.5)	77.8 (58.3–89.7)	100.0	91.3 (79.2–97.6)
IgG 18 g/L	STP in normohydrated calves	18	75 (42.8–94.5)	100.0 (54.1–100.0)	100.0	66.7 (42.9–84.2)	83.3 (58.6–96.4)
	STP in dehydrated calves	54	74.3 (56.7–87.5)	63.2 (38.4–83.7)	78.8 (66.6–87.4)	57.1 (40.8–72.1)	70.4 (56.4–82.0)
	GGT in calves with age > 3 gg	61	70.3 (53.0 -84.1)	87.5 (67.6–97.3)	89.7 (74.7–96.2)	65.6 (53.2–76.2)	77.1 (64.5–86.9)
	GGT in normohydrated calves > 3 days	15	66.7 (29.9–92.5)	100.0 (54.1–100)	100.0	66.7 (44.3–83.4)	80.0 (51.9–95.7)
	GGT in dehydrated calves > 3 days	46	71.43 (51.3–86.8)	83.3 (58.6–96.4)	87.0 (69.8–95.1)	65.2 (50.2–77.7)	76.1 (61.2–87.4)
IgG 25 g/L	STP in normohydrated calves	18	100.0 (79.4–100.0)	100.0 (15.8–100.0)	100.0	100.0	100.0 (81.5–100.0)
	STP in dehydrated calves	54	93.0 (80.9–98.5)	45.5 (16.7–76.6)	87.0 (79.4–92.0)	62.5 (31.9–85.6)	83.3 (70.7–92.1)
	GGT in calves with age > 3 gg	61	58.3 (43.2–72.4)	92.3 (64.0 -99.8)	96.6 (80.8–99.5)	37.5 (29.3–46.5)	65.6 (52.3–77.3)
	GGT in normohydrated calves > 3 days	15	61.5 (31.6–86.1)	100 (15.8–100)	100	28.6 (16.7–44.3)	66.7 (38.4–88.2)
	GGT in dehydrated calves > 3 days	46	62.9 (44.9–78.5)	90.9 (58.7–99.8)	95.7 (76.9–99.3)	43.5 (32.5–55.2)	69.6 (54.3–82.3)

N, number of calves; Se, sensitivity; Sp, specificity; PPV, positive predictive value; NPV, negative predictive value; CI 95%, 95% confidence interval; Acc, accuracy

## Discussion

The main finding of this study was that serum GGT activity was correlated with IgG concentration even in diarrheic calves with clinical dehydration with higher test performance than STP. Our findings agree with those reported by Tyler et al. (1999) and Fecteau and colleagues (2013) and can be explained by the fact that dehydration weakly affects serum GGT activity. Regarding the correlation between IgG and GGT, age was the factor that showed a significant influence. This result supports evidence from previous observations that age significantly alters the relationship between IgG concentrations and GGT activity (Braun et al. 1982; Cuttance et al. 2019; Mugnier et al. 2020). Similarly, in sick calves, Fecteau et al. (2013) found that age significantly affected the prediction of serum IgG concentration using GGT activity in sick calves aged 1 to 13 days, but the inclusion of age in the prediction model had a minor impact on the results. On the other hand, the prediction of serum IgG concentration based on GGT activity in beef calves generated a more accurate model when the study population was limited to calves less than 8 days old compared to those 18 days old (Wilson et al. 1999). Also, in our study, it is interesting to note that Spearman’s coefficient in the whole sample (calves aged between 1 and 10 days) was 0.63, but it was higher when considering only calves older than 3 days (0.71).

The greater correlation in calves aged > 3 days shown in our study can be explained by the fact that serum GGT activity tends to stabilize 24–48 h after colostrum ingestion and then slowly decrease for about 20 days (Braun et al. 1982). Therefore, we argue that selecting calves between 3 and 10 days old reduces the age-related variability of enzyme activity, thereby increasing the diagnostic efficiency of FTPI in diarrheic calves.

Serum STP was less correlated to serum IgG concentration than GGT activity in dehydrated calves. This result was evidenced by the difference in diagnostic accuracy found in sick calves with or without dehydration signs (see Tables 2 and 3). This finding was expected and could be attributed to the influence of fluid loss on STP measurement. (Buczinski et al. 2018; Tyler et al. 1999). Another source of uncertainty in predicting FTPI in diarrheic calves by serum refractometry could be the hypoproteinemia, which can affect the evaluation due to gastrointestinal loss or malnutrition (Bartlett et al. 2006; Athanasiou et al. 2019). Therefore, the use of serum refractometry to estimate FTPI in dehydrated calves should be discouraged because it has many limitations and variables that could bias the assessment.

Our results have some clinical applications. The GGT activity was more correlated with serum IgG concentrations in calves over 3 days old than STP in dehydrated calves. Although this method cannot be commonly used in field conditions, one possible use could be related to assessing passive immunity, for example, during an NCD outbreak involving many calves, because this method is inexpensive and easy to perform in any laboratory. This result could help practitioners collect accurate prognostic information on sick calves and on-farm colostrum management performance when the serum refractometer does not offer the utility it has in healthy calves or otherwise nondehydrated ones.

The main limitation of this study was that the retrospective analysis of diarrheic calves was carried out in a hospital setting where hospitalization involves animals from different herds with different clinical signs or where treatments have frequently been performed and had no effect. Such differences were not reported in this study; therefore, we cannot exclude that they affected the results and the external validity of the findings, especially regarding the magnitude of milk feeding before hospitalization or colostrum management practices of each farm, which may have affected all biomarkers considered in our study. However, GGT activity has shown robust diagnostic ability in dehydrated calves compared to STP and, in our opinion, could also have good results in the field or in single herds where more standardized calf management conditions are usually available.

In conclusion, serum GGT activity at an optimal cutoff point of 100 U/L showed satisfactory performance predicting the cutoff of 10 g/L IgG in dehydrated diarrheic calves between 3 and 10 days after birth. The accuracy of the STP refractometer was lower than GGT activity in dehydrated calves. Data from this study confirm and expand other reports that hypothesized the superiority of GGT activity in diagnosing FTPI for dehydrated calves. On the other hand, the accuracy of the two diagnostic methods under examination was similar in normohydrated diarrheic calves, making GGT activity disadvantageous in these calves because of both more significant difficulties in obtaining the data under field conditions compared with a refractometer and the more noticeable influence of age in correlating with IgG.

### Electronic supplementary material

Below is the link to the electronic supplementary material.

Supplementary Material 1

## Data Availability

The data that support the findings of this study are available on request from the corresponding author A.B.
