# Supplementary material for: Assessing failure of transfer of passive immunity by gamma-glutamyl-transferase activity and serum refractometry in holstein-friesian calves affected by neonatal diarrhea
Source: Vet Res Commun. 2023 Jun 14;47(4):2315–21. doi: 10.1007/s11259-023-10149-3 (PMC10698073; doi:10.1007/s11259-023-10149-3)

**Veterinary Research Communication**

**Supplementary files**

**Assessing failure of transfer of passive immunity by gamma-glutamyl-transferase activity and serum refractometry in Holstein-Friesian calves affected by neonatal diarrhea**

Giulia Sala ^1†^, Valerio Bronzo ^1†^, Antonio Boccardo ^1*^, Alessia Libera Gazzonis ^1^, Pierangelo Moretti ^1^, Vincenzo Ferrulli ^1^, Angelo Giovanni Belloli ^1^, Laura Filippone Pavesi ^1^, Gaia Pesenti Rossi ^1^ and Davide Pravettoni ^1^

^1^ Department of Veterinary Medicine and Animal Science, University of Milan, Via dell’Università 6, 26900 Lodi, Italy.

^*^Corresponding author: antonio.boccardo@unimi.it

^†^ These authors contributed equally to this work.

**Supplementary Table 1:** Median (1st and 3rd quartile) of immunoglobulin G (IgG), serum total protein (STP), gamma-glutamyl-transferase (GGT), and age in 91 Holstein calves included in the study. Of these, 72 calves were affected by neonatal calf diarrhea (NCD), while 19 were healthy.

|  | **Number** | **IgG (g/L)*** | **STP (g/L)*** | **GGT (IU/L)** | **Age (days)*** |
| --- | --- | --- | --- | --- | --- |
| *Healthy calves* | 19 | 25.4 (18.9-28.6) | 54 (52-58) | 234 (106-525) | 7 (4-8) |
| *Calves with NCD* | 72 | 14.2 (9.4-21.9) | 60 (52-68) | 158 (84.5 -343) | 8 (5-10) |

**P* < 0.05

**Supplementary Table 2.** Median (1st and 3rd quartile) of immunoglobulin G (IgG), serum total protein (STP), gamma-glutamyl-transferase (GGT) in calves in 72 diarrheic Holstein Friesian calves examined at a referral university hospital according to the dehydration degree.

|  | **Number of calves** | **IgG (g/L)** | **STP(g/L)** | **GGT (IU/L)** |
| --- | --- | --- | --- | --- |
| *Normohydrated* | 18 | 14.7 (9.7-22) | 58 (52-60) | 164.5 (95-246) |
| *Dehydration of 3-5%* | 20 | 13.3 (8.7-19.2) | 59 (48-60) | 151 (95-296.5) |
| *Dehydration of 6-8%* | 27 | 17.8 (11.2 -26) | 66 (56 -74) | 172 (72-417) |
| *Dehydration of* ≥*9%* | 7 | 10.6 (9.4-16.8) | 70 (48-78) | 135 (30-360) |

The dehydration score was estimated according to the following scoring system:

Normohydrated calves, upper eyelid skin tent <2 s;

Estimated loss of body mass 3–5%, eyeball slightly sunken (1–2 mm), and upper eyelid skin tent >2 s but <4 s;

Estimated loss of body mass 6–8%, sunken eyes (3–4 mm), dry nose, upper eyelid skin tent >5 s;

Estimated loss of body mass ≥9%, severe sunken eyes with an easily perceptible distance between the eyeball and the eyelid (≥5 mm), cold ears, legs, and oral cavity, dry mouth, and nose, upper eyelid skin tent persist

**Supplementary Table 3.** Contingency table representing the number of calves and percentage of 72 calves affected by neonatal calf diarrhea (NCD) and 19 healthy calves categorized by consensus category of serum immunoglobulin G (IgG) concentration for the evaluation of passive immunity in dairy calves proposed by Lombard et al. (2020)^1^.

|  | *Healthy calves* | *Diarrheic calves* | *tot* |
| --- | --- | --- | --- |
| *Poor (IgG < 10 g/L)* | 2 | 23 | 25 |
|  | 8% | 92% | 100% |
| *Fair (IgG: 10-17.9 g/L)* | 1 | 22 | 23 |
|  | 4.4% | 95.6% | 100% |
| *Good (IgG: 18-24.9 g/L)* | 6 | 14 | 20 |
|  | 30% | 70% | 100% |
| *Excellent (IgG > 25 g/L)* | 10 | 13 | 23 |
|  | 43.5% | 56.5% | 100% |

^1^ Lombard, J., Urie, N., Garry, F., Godden, S., Quigley, J., Earleywine, T., ... & Sterner, K. (2020). Consensus recommendations on calf-and herd-level passive immunity in dairy calves in the United States. *Journal of dairy science*, *103*(8), 7611-7624.

**Supplementary Table 4.** Median (1st and 3rd quartile) of gamma-glutamyl transferase (GGT) and serum total protein (STP) in the different immunoglobulin G (IgG) classes.

|  | **GGT (IU/L)** | **STP (g/L)** |
| --- | --- | --- |
| *Poor (IgG < 10 g/L)* | 81 (43-100)^a^ | 47 (43-56)^a^ |
| *Fair (IgG: 10-17.9 g/L)* | 189 (135-360)^b^ | 60 (52-65)^b^ |
| *Good (IgG: 18-24.9 g/L)* | 235 (129-303)^b^ | 60 (55-67)^b^ |
| *Excellent (IgG > 25 g/L)* | 339 (167-577)^b^ | 60 (58-72)^b^ |

For each category with different superscript letters (a, b) are statistically different from each other (p−value < 0.05, multiple comparison), while those with the same superscript letters (a, b) are not statistically different from each other (p−value > 0.05, multiple comparison).

**Fig. 1** Box plot of serum immunoglobulin G (IgG) concentration, serum total protein (STP), and gamma-glutamyl-transferase (GGT) activity in 72 calves affected by neonatal calf diarrhea (NCD) and 19 healthy


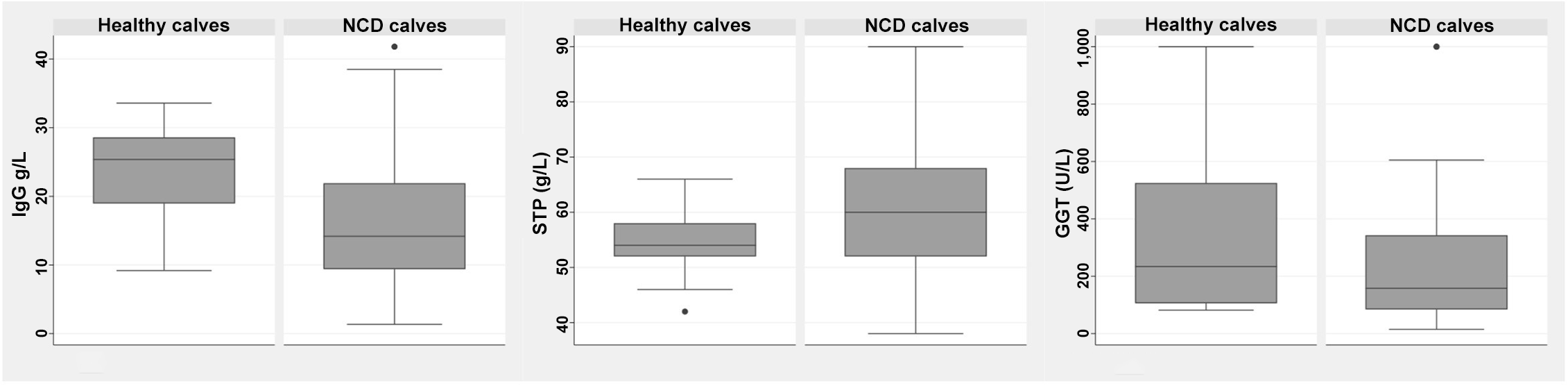


**Fig. 2** Receiver Operating Characteristic (ROC) curves for optimal serum total protein (STP) cut-offs, in 72 calves with neonatal calf diarrhea. The gold standard used was immunoglobulin G (IgG) concentration. In figures A and B, the cut-off of the gold standard is set at 10 g/L. In figures C and D, the cut-off of the gold standard is set at 18 g/L; finally, in figures E and F, the cut-off of the gold standard is set at 25 g/L.

**
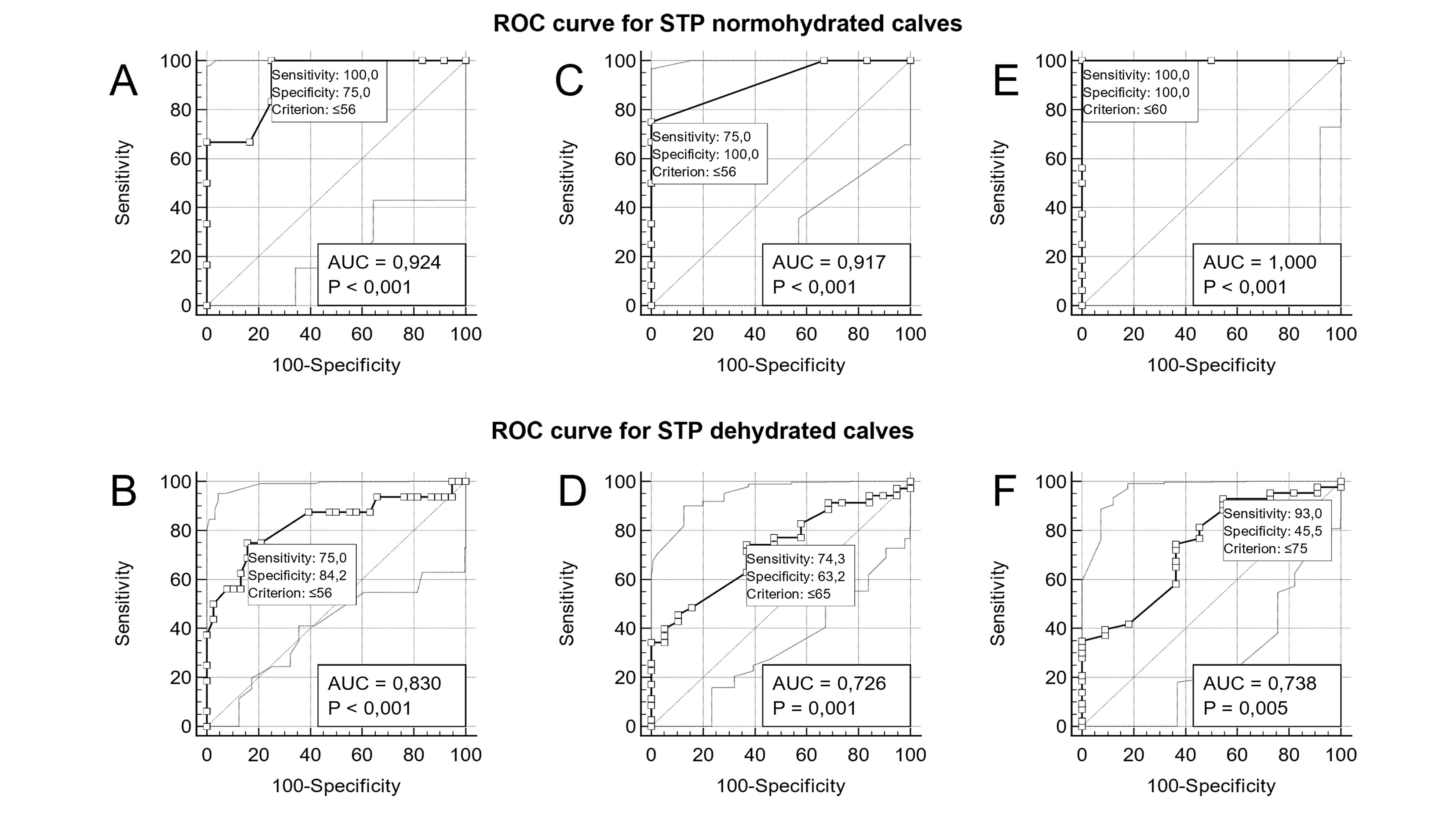
**

**Fig. 3** Receiver Operating Characteristic (ROC) curves for determination of optimal gamma-glutamyl-transferase (GGT) cut-offs in 72 calves with neonatal calf diarrhea. The gold standard used was immunoglobulin G (IgG) concentration. In image A, the cut-off of the gold standard is set at 10 g/L. In image B, the cut-off of the gold standard is set at 18 g/L; finally, in image C, the cut-off of the gold standard is set at 25 g/L. The ROC curves of GGT activity were constructed only for animals older than three days because there were only 11 animals under 3 days old.


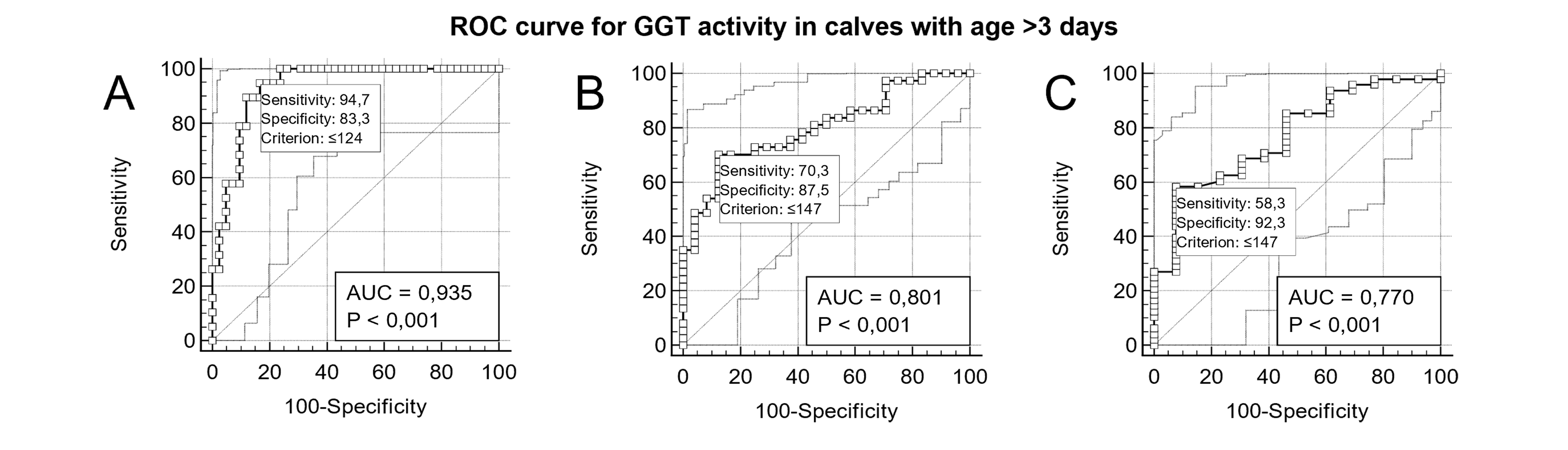

Supplement: Supplementary file 1 — Supplementary Material 1 [file 11259_2023_10149_MOESM1_ESM.docx]
